# Properties of Renshaw-like cells excited by recurrent collaterals of pudendal motoneurons in the cat

**DOI:** 10.1186/s12576-020-00763-0

**Published:** 2020-07-13

**Authors:** Ken Muramatsu, Masatoshi Niwa, Sei-Ichi Sasaki

**Affiliations:** 1 Department of Physical Therapy, Kyorin University, 5-4-1 Shimorenjaku, Mitaka, Tokyo 181-8612 Japan; 2 Department of Occupational Therapy, Kyorin University, 5-4-1 Shimorenjaku, Mitaka, Tokyo 181-8612 Japan; 3 Center for Medical Sciences, Ibaraki Prefectural University of Health Sciences, 4669-2 Ami, Ami-machi, Inashiki, Ibaraki 300-0394 Japan; 4 Present Address: Toyo Public Health College, 6-21-7 Hommachi, Shibuya-ku, Tokyo 151-0071 Japan

**Keywords:** Pudendal motoneurons, Recurrent collateral, Renshaw cell, Interneuron, Recurrent inhibition

## Abstract

Although anatomical studies have indicated pudendal motoneurons to give off recurrent collaterals, they are not considered to make synapses onto interneurons, such as Renshaw cells, and rather terminate their own signals. No study till date has examined interneurons being driven by recurrent collaterals of pudendal motoneurons. Here, we aimed to investigate the existence of Renshaw cells driven by pudendal motoneurons along with the recurrent inhibition of the latter. Extracellular recordings were obtained from the ventral horn of the sacral spinal cord of anesthetized cats. Dorsal roots were sectioned, and motor axons were electrically stimulated. Renshaw-like cells driven by recurrent collaterals, with high-frequency firings at short latency discharge, were observed around Onuf’s nucleus. However, the recurrent inhibitory post-synaptic potentials were not recorded by intracellular recordings from the pudendal motoneurons. In summary, we found Renshaw-like cells driven by pudendal motoneurons, but we could not identify the synaptic connection of these neurons.

## Background

The pudendal motoneurons (PMNs) are located in Onuf’s nucleus, innervating perineal striated muscles, such as external anal and urethral sphincters, and the ischiocavernosus and bulbospongiosus muscles [1, 2]. These muscles contribute to urinary continence, fecal continence, and sexual function by the interplay of autonomic nervous system [3, 4].

The present study focused on the connection of recurrent collaterals of PMNs. Recurrent collaterals of MNs are known to make synapses on inhibitory interneurons named Renshaw cells (RCs) [5]. Recurrent inhibitions (RIs) of MNs are mediated by these RCs. RIs modulate not only MN activity but also suppress certain excitatory synaptic input to MNs [6, 7]. Thereby, the connection of recurrent collaterals of MNs is important to understand motor control of pelvic floor muscles. Although anatomical studies had indicated PMNs to also give off recurrent collaterals, they were not considered to make synapses on RCs, and rather terminated the signals themselves [8]. Previous studies had reported recurrent inhibitions (RIs) mediated by RCs to be absent in PMNs [9, 10]. There, however, could be a possibility of weak RI associated with PMNs, since small recurrent inhibitory post-synaptic potentials (IPSPs) were observable when averaging techniques were used. Because previous studies have indicated that about 100-μV weak RIs can be detected using averaging techniques [5, 11], in this study, we investigated whether (i) RCs driven by PMNs exist, and (ii) RIs occur in PMNs.

## Methods

### General procedures

All experimental procedures were approved by the Animal Ethics Committee of Ibaraki Prefectural University of Health Sciences and were in accordance with the guiding principles for care and use of animals in the field of physiological sciences outlined by the Physiological Society of Japan. Fourteen Male and female adult cats (Shiraishi Animals, Koshigaya, Japan) weighing 2.8–4.6 kg were anesthetized with an i.p. injection of sodium pentobarbital (35–40 mg/kg). The femoral artery and forearm vein were cannulated, and arterial blood pressure was maintained at 100–130 mmHg via intravenous administration of appropriate amounts of pressor agents (Noradrenalin, Daiichi-Sankyo, Tokyo, Japan). Deep aesthesia (narrow pupil size and stable arterial blood pressure) was subsequently maintained using supplemental doses of sodium pentobarbital throughout the experiments (4–7 mg/kg/h, i.v.). The animals were then immobilized with pancuronium bromide and ventilated artificially. The end-tidal CO_2_ and blood pressure were continuously monitored and body temperature maintained at 37 °C with a heating sheet.

**Fig. 1:**
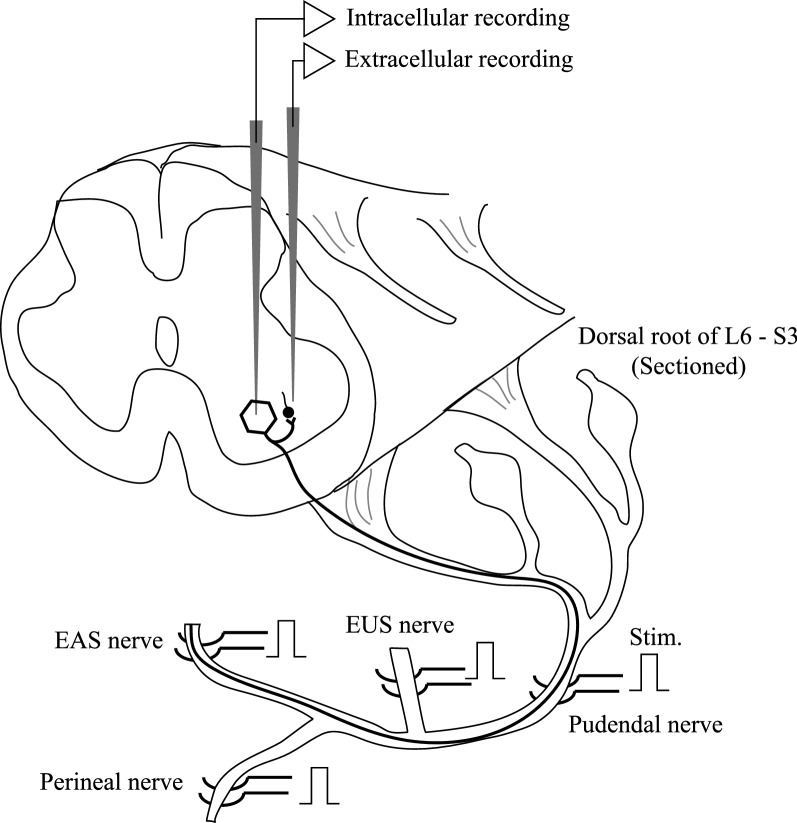
Experimental arrangement. The dorsal roots were cut to record extracellular spikes of single interneurons excited by recurrent collaterals of PMNs and recurrent-IPSPs. EAS nerve, muscle nerve of the external anal sphincter muscle; EUS nerve, muscle nerve of the external urethral sphincter muscle. Perineal nerve innervates the bulbospongiosus and ischiocavernosus muscle. Stim., stimulation

The animals were placed in a holder, laminectomy was performed from the L5 to S3 vertebrae, and the dorsal roots of L6 to S3 were bilaterally sectioned. Subsequently, the pudendal nerve, muscle nerve of the external anal sphincter muscle (EAS nerve), muscle nerve of the external urethral sphincter muscle (EUS nerve), and perineal nerve that innervate the bulbospongiosus and ischiocavernosus muscle were mounted on a bipolar silver-hook electrode for stimulation. Nerves were stimulated with rectangular pulses of 150 μs in duration. The preparation described above allows only the antidromic volley in the motor nerve fibers to enter the spinal cord when the central stump of the pudendal nerve is stimulated. All exposed tissues were covered with a pool of paraffin oil kept at 37 °C (Fig. 1). Seven cats were used for mapping of RCs and others were used for intracellular recording of PMNs.

### Mapping of interneurons

For recording spikes of interneurons that were driven by recurrent collaterals of PMNs, extracellular recordings from the ventral horn of the S1 to S2 region were made using glass-capillary microelectrode filled with Fast Green FCF dye in 3 M NaCl. The minimum strength of stimulation evoking field potentials of PMNs was defined as threshold. Discharge of interneurons was recorded during gradual increase of the strength of stimulation come down to evoke maximal amplitude of antidromically field potential of PMNs. When each branch of pudendal nerve was able to stimulate separately, in addition to the above manipulation, we stimulated each branch of the pudendal nerve to test their convergence to interneurons from other muscle nerves. Single unit’s discharge was recorded using a data recorder (PC208AXx, SONY, Tokyo, Japan) and analyzed using PowerLab/8 s (ADInstruments, Dunedin, New Zealand). After a cell was identified and its physiological characteristics examined, a negative current of 20 µA was passed through the electrode for 15 min and recording site marked by Fast Green FCF dye.

After the experiment, the animals were deeply anesthetized and perfused transcardially with 10% formalin solution. Next, the spinal cord from L7 to S3 was removed and was serially and transversally sectioned at 100 µm on a freezing microtome. Subsequently, sections were mounted on gelatinized glass slides, stained with Cresyl violet, and examined using bright field light microscopy. In case of successful staining, a small intense green spot could be seen in the ventral horn of spinal cord.

### Intracellular recording of PMNs

Intracellular recording from antidromically identified MNs was obtained with glass-capillary micro-electrodes filled with 2 M K-citrate. To examine recurrent IPSPs, the corresponding pudendal nerve was stimulated at an intensity just sub-threshold for antidromic excitation of the impaled cell, and the record was averaged over 200 times. Subsequently, the electrode was slightly moved to the outside of the impaled cell and extracellular recording was performed in the same manner as intracellular recording. Records were stored in a data recorder (PC208AX, SONY) and analyzed using PowerLab/8 s (AD Instruments). Data are presented as mean ± S.D.

## Results

As described below, we found that the interneurons are driven by recurrent collateral of PMNs. Regardless of firing properties and the fact that these interneurons resemble RCs, we could not record recurrent IPSPs from PMNs. Therefore, it cannot be concluded that these interneurons are RCs. For convenience, we call these interneurons Renshaw-like cells (RLCs) below.

### Physiological properties of RLCs

**Fig. 2:**
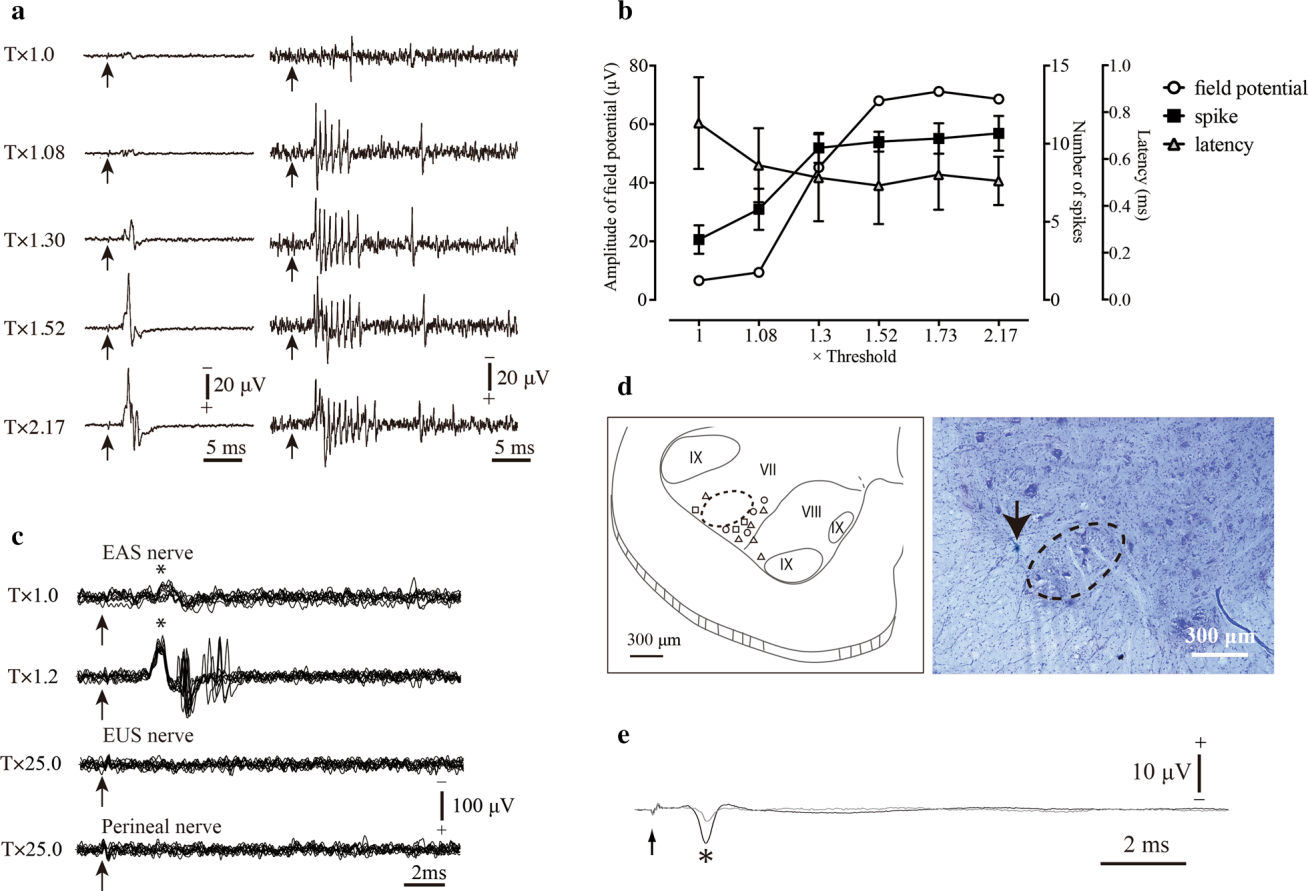
Representative response of RLCs and intracellular recording from PMNs. **a** The antidromic field potential of pudendal MNs (left) and discharges of RLCs in S1 spinal level, which were excited by stimulation of the pudendal nerve (right). Discharge was recorded while gradually increasing the strength of stimulation to 2.17 × threshold in this case. The arrow indicates stimulus artifact. **b** A typical relationship among stimulus intensity, number and latency of the activated spikes, and amplitude of the field potentials. The circle represents the amplitude of antidromic field potential. The square indicates the number of activated spikes. The triangle indicates latency of the first spikes of RLCs. The abscissa indicates the intensity of stimulation multiplied by threshold of the stimulus voltage. The firing frequency of RLCs gradually increased and latency of first spike of RCs gradually decreased according to stimulation intensity, reaching plateaus at intensities sub-maximal of antidromic volley (1.3 × of threshold in this case). **c** Convergence of excitatory input from other pudendal nerves. The EAS nerve, EUS nerve, and perineal nerves were stimulated. Note that the RLC was activated only when EAS nerve was stimulated. All waves were superimposed 10 to 15 times. The arrow indicates stimulus artifact. The asterisk indicates antidoromic field potential of the PMNs. **d** Synthetograph of each RLC, showing the anatomical relationship between Onuf’s nucleus (dotted circle) and RLCs. The circle represents the recording site of RLCs activated by stimulation of the pudendal nerve; the square indicates that by the EAS nerve; and the triangle indicates that by the EUS nerve. **e** Intracellular recording from EAS motoneurons (following the stimulation of the EAS nerve, at intensities just below the threshold for activation of antidromic spikes in the impaled EAS motoneurons). Although membrane potentials were summed over 200 times, synaptic potentials were not observed. The arrow indicates stimulus artifact. The asterisk indicates antidoromic field potential of the PMNs. The gray trace indicates extracellular recording and the black trace indicates intracellular recording

We recorded the discharge of 13 RLCs which were activated by axon collaterals of PMNs from 7 animals. Discharges from these RLCs were recorded from ventral horn of S1 vertebral level. Four of the RLCs were excited by stimulation of the whole pudendal nerve, three were excited by stimulation of the EAS nerve, and six were excited by stimulation of the EUS nerve. The thresholds of orthodromic activation of RLCs were approximately same or just above the threshold of antidromic field potential, and these RLCs did not show tonic background firing (Fig. 2a, c).

The latencies of onset of the first spike of RLC from the onset of antidromic field potentials showed a tendency to shorten according to increasing stimulus strength (Fig. 2b). At maximal stimulation, the value of the latencies ranged around 0.5–2.62 ms. The initial frequency of discharge of RLCs, measured from the interval between first and second spikes, was highest for the first impulses and gradually declined thereafter (Fig. 2a). The initial frequency was 905 ± 194.2/s on average. The number of maximal spikes, however, varied across the neurons. Some RLCs showed only 2–3 spikes at maximal stimulation while others showed 5–8 spikes. On an average, the number of spikes was 2.3 ± 1.7. The duration of discharges also varied over 1.6–15.4 ms. In general, RLCs fired for 4–5 ms.

RLCs that received recurrent excitatory collateral from EAS or EUS MNs were not fired when other muscle nerves were stimulated at supra-maximal intensity of antidromic volley (Fig. 2c).

### Recording sites of RLCs

We recorded the discharge of 13 RLCs activated by axon collaterals of PMNs from 7 animals. The photograph in Fig. 2d shows the typical appearance of green spots. These spots were distinctly different from that in other tissues, and hence, easily identified. A synthetograph showing the anatomical relationship between Onuf’s nucleus and RLCs is shown in Fig. 2d. All the RLCs were found very close to Onuf’s nucleus while no RLC was found inside the nucleus. Eleven of 11 RLCs were found in the medial sites of Onuf’s nucleus and only 2 were found in the lateral sites of Onuf’s nucleus.

### Intracellular recording of PMNs

Obtaining stable intracellular recording from PMNs has been more difficult than from hindlimb MNs because of the smaller size of cell bodies [12]. Therefore, we could obtain intracellular recordings without obvious damages, at membrane potential more negative than 40 mV, with no spontaneous firing of action potentials, and summed signals over 200 times in only 10 out of 22 MNs. These 10 MNs (EAS MNs: 6, EUS MNs: 4) which complied with the standards as mentioned above were used for analysis. The membrane potential of these MNs was 46.2 ± 5.12 mV. Following stimulation of the EAS or EUS nerve at an intensity just below the threshold for activation of antidromic spikes in the impaled MNs, only the noise level fluctuation of the membrane potential, a few micro-volts or less, was observed. We could not find any synaptic potentials from these MNs. Even if we included the 12 discarded MNs for data analysis, there was no synaptic potential (Fig. 2e).

## Discussion

In the present study, RLCs that were synaptically activated by recurrent collaterals of PMNs were found around Onuf’s nucleus. Hindlimb RCs are well known to deliver a high-frequency burst with an initial rate of approximately 1000/s and a duration of about 40 ms [5]. In the same manner, RLCs in the present study fired with an initial rate of approximately 900/s. While the duration of spike discharge was much shorter than in hindlimb RCs, it was similar to that in RCs driven by abdominal and intercostal MNs [5, 13, 14]. The latencies of the onset of the first spike of RLC from the onset of antidromic activations ranged over 0.5–2.62 ms, suggesting the spikes to have been activated mono-synaptically in the shortest pathway, same as in hindlimb RCs [5].

The recording site of RLCs was frequently seen in a medial part of Onuf’s nucleus and less commonly found in a lateral part. Results were in accordance with the fact that the buttons of axon collaterals of pudendal MNs are rich in the medial sites of Onuf’s nucleus and scarce in the lateral sites [8]. It is still not clear whether RLCs were located in the inside of Onuf’s nucleus, since field potentials of PMNs might have hidden the spikes of RLCs. The number of recorded RLCs in each animal was very small compared to the buttons of axon collaterals previously reported [8]. This suggested only a small number of collaterals to terminate the RLCs, most terminating on PMNs themselves, as predicted previously [8]. Interestingly, there was no convergence to RLCs from other muscle nerves, thereby suggesting the activities of these RLCs to be highly reflecting the motor output of MNs innervating a particular muscle; the functional significance of such connection, however, still remains unclear.

Surprisingly, RIs were absent in PMNs, although we averaged the membrane potentials over 200 times. The results were in accordance with previous studies, although the observations did not use averaging technique [9, 10]; small recurrent IPSPs are known to be observable only when averaging techniques were used [11]. Hence, our observation indicated most PMNs to not receive even weak RIs from RLCs. However, whether PMNs completely lack RIs could not be concluded fully, since a small number of PMNs might receive weak RIs, as reported recently in abdominal MNs [13]. Collectively, existence of RIs in PMNs remains debatable.

MNs innervating the limb muscles in cats are known to exhibit strong RI pathways, which weaken in the trunk muscle [5, 13, 14]. Especially respiratory MNs and abdominal MNs exhibit weak recurrent inhibition [11, 13, 14]. It is possible that RIs in trunk muscle become weaker as one moves from the outside to the center of the muscles, and disappear eventually in pelvic floor muscle. In other words, target cells of these RLCs may be different from typical RCs. If so, it becomes significant to know where the RLCs connect. However, we could not address this point in the present study, and would highlight the need for further studies in future.

The present study possesses some notable limitations. First, there is a possibility of bias in the selection of MNs for recordings; the MNs from which we obtained intracellular recordings may have been larger PMNs because of the difficulty in obtaining stable intracellular recordings from smaller PMNs. We might not be able to test recurrent IPSPs in smaller PMNs. Second, all experiments were performed with the subjects under anesthesia, and it remains unknown how RCs behave during wakefulness. Analysis using electrophysiological techniques, however, is nearing its limits because of the difficulty discussed above. Therefore, in the future, it is needed to proceed with research not only using electrophysiological methods but also by combining histological methods such as immunohistochemical analysis [15].

## Conclusions

In summary, the present study indicated recurrent collaterals of PMNs to project RLCs. This presented a new aspect of the termination of recurrent collaterals of PMNs, since motor axon collaterals of PMNs had been previously predicted to make synaptic connection with PMNs only [6]. However, we could not identify the synaptic connection of these neurons in this study. Further studies are warranted to clarify the target neuron and synaptic nature of these RLCs and understand the functional significance of these neurons.

## Data Availability

Not applicable.
